# Cationic Amino Acid Transporter-1-Mediated Arginine Uptake Is Essential for Chronic Lymphocytic Leukemia Cell Proliferation and Viability

**DOI:** 10.3389/fonc.2019.01268

**Published:** 2019-11-20

**Authors:** Anke Werner, Daniel Pieh, Hakim Echchannaoui, Johanna Rupp, Krishnaraj Rajalingam, Matthias Theobald, Ellen I. Closs, Markus Munder

**Affiliations:** ^1^Third Department of Medicine (Hematology, Oncology, and Pneumology), University Medical Center of the Johannes Gutenberg University Mainz, Mainz, Germany; ^2^Department of Pharmacology, University Medical Center of the Johannes Gutenberg University Mainz, Mainz, Germany; ^3^Cell Biology Unit, University Medical Center of the Johannes Gutenberg University Mainz, Mainz, Germany; ^4^Research Center for Immune Therapy, University Medical Center of the Johannes Gutenberg University Mainz, Mainz, Germany; ^5^German Cancer Consortium (DKTK), Mainz, Germany; ^6^German Cancer Research Center (DKFZ), Heidelberg, Germany

**Keywords:** tumor metabolism, arginine, amino acid transporter, chronic lymphocytic leukemia, nutrient restriction

## Abstract

Interfering with tumor metabolism by specifically restricting the availability of extracellular nutrients is a rapidly emerging field of cancer research. A variety of tumor entities depend on the uptake of the amino acid arginine since they have lost the ability to synthesize it endogenously, that is they do not express the rate limiting enzyme for arginine synthesis, argininosuccinate synthase (ASS). Arginine transport through the plasma membrane of mammalian cells is mediated by eight different transporters that belong to two solute carrier (SLC) families. In the present study we found that the proliferation of primary as well as immortalized chronic lymphocytic leukemia (CLL) cells depends on the availability of extracellular arginine and that primary CLL cells do not express ASS and are therefore arginine-auxotrophic. The cationic amino acid transporter-1 (CAT-1) was the only arginine importer expressed in CLL cells. Lentiviral-mediated downregulation of the CAT-1 transporter in HG3 CLL cells significantly reduced arginine uptake, abolished cell proliferation and impaired cell viability. In a murine CLL xenograft model, tumor growth was significantly suppressed upon induced downregulation of CAT-1 in the CLL cells. Our results suggest that inhibition of CAT-1 is a promising new therapeutic approach for CLL.

## Introduction

Chronic lymphocytic leukemia (CLL) is the most common leukemia in the Western World. It is characterized by the accumulation of mature, typically CD5^+^CD23^+^ neoplastic B-lymphocytes in the peripheral blood, lymph nodes, bone marrow and/or other lymphoid organs (1). Despite tremendous progress in treatment options by B cell receptor signal transduction inhibitors (ibrutinib, idelalisib) and bcl2 inhibitors (venetoclax), it still remains an incurable disease that needs novel treatment approaches.

One of the key hallmarks of a cancer cell is its metabolic reprogramming that supports proliferation and acquisition of biomass (2). This offers the possibility to interfere therapeutically with non-redundantly up- or downregulated branches of the rewired cancer cell metabolism (3, 4). The increased demand of cancer cells for nutrients is apparent in the well-established enhanced uptake of glucose in conjunction with its preferential use via aerobic glycolysis, referred to as the Warburg effect (5). Amino acids are another important class of nutrients, which cancer cells need to acquire and metabolize for viability and continuous growth (5, 6). Asparagine depletion via asparaginase in acute lymphoblastic leukemia (ALL) is an example for interference with amino acid availability as clinically established anti-tumor strategy. Cysteine depletion by cysteinase is in preclinical development for CLL (7) based on the inherent dependency of CLL cells on cysteine for glutathione synthesis (8).

Arginine, a semi-essential proteinogenic amino acid that becomes limiting only in situations of increased demand, is a substrate for metabolic pathways leading to e.g., nitric oxide (NO), polyamines and collagen (9, 10), and is also a key activator of mammalian target of rapamycin (mTOR), a crucial metabolic checkpoint of cellular proliferation (11). The availability of arginine is essential for the proliferation and function of physiological immune cells (12, 13) and is also a decisive regulatory parameter within the tumor microenvironment: both, tumor cells and immune cells compete for this amino acid (13–16). Most tissues constitutively or inducibly express the enzymes argininosuccinate synthase (ASS) and argininosuccinate lyase (ASL), which metabolize the non-proteinogenic amino acid citrulline to arginine in a two-step reaction (17, 18). In contrast, various tumor entities do not express ASS and therefore depend on the availability and uptake of extracellular arginine. This so-called arginine-auxotrophy (19) has been shown for solid cancer entities (e.g., melanoma, sarcoma, hepatocellular carcinoma) (20), but has more recently also been described for hematological cancers like acute myeloid leukemia (21) or acute lymphoblastic leukemia (22). Restricting the availability of arginine by the systemic administration of the arginine-metabolizing enzymes arginine deiminase (ADI) (23, 24) or arginase (25) is already in clinical development with promising results.

A novel therapeutic strategy to deprive cancer cells of arginine would be to interfere with its uptake (5) if (i) a defined arginine transporter would be overexpressed in a tumor entity compared to physiological tissues and more importantly, (ii) restriction of the availability of arginine upon blockade of this transporter could be compensated by physiological tissues as opposed to cancer cells (26). Arginine transport through the plasma membrane of mammalian cells is mediated by eight different transporters, with different tissue distribution (27, 28). The cationic amino acid transporters (CAT-1, CAT-2A, CAT-2B, and CAT-3; SLC7A1-3) recognize exclusively cationic amino acids as their substrates, while the heteromeric amino acid transporters y^+^LAT1 (SLC7A7), y^+^LAT2 (SLC7A6), and b^0,+^AT (SLC7A9), as well as ATB^0,+^ (SLC6A14) also transport neutral amino acids (27, 29). CATs can mediate unidirectional arginine transport into cells (27, 28) and are the main uptake transporters in most mammalian cells. In contrast, y^+^LATs are obligatory exchangers. They catalyze primarily arginine export in exchange with neutral amino acids and sodium under physiological conditions (27, 28).

Studies on amino acid transporters in cancer have so far mainly focused on glutamine transport mediated by ATB^0,+^ (30) as well as the exclusive neutral amino acid transporters ASCT2 (SLC1A5) (31, 32) and LAT1 (SLC7A5) (33) showing a dependence of cancer cell growth on transporter-mediated amino acid uptake. However, the expression, regulation and potential non-redundant function of defined arginine transporters for cancer cell viability and growth has not been studied so far in hematological cancer entities. Here we analyzed the expression of arginine transporters in human primary CLL cells as well as CLL cell lines. We found a non-redundant role of the arginine transporter CAT-1 for CLL cell viability and proliferation. Our data address previously unrecognized aspects of CLL cell physiology and—more importantly—identify a potential novel therapeutic target structure for CLL therapy.

## Materials and Methods

### Reagents

Unless mentioned otherwise, reagents were purchased from Roth (Karlsruhe, Germany) or Sigma Aldrich (Steinheim, Germany). Amino acids were L-isomers.

### Primary Cells and Cell Lines

This study was approved by Rhineland-Palatinate Medical Association Ethics Committee. Blood donors gave written informed consent in accordance with the Declaration of Helsinki. Primary CLL cells were isolated from peripheral blood of CLL patients (Supplementary Table S1) by Ficoll density gradient centrifugation and harvested from the interphase (fraction of CD5^+^CD19^+^ cells > 95% within the mononuclear cell population in all cases). Activation of CLL cells (6 × 10^6^/ml) was induced by 7.5 μg/ml Toll-like receptor (TLR)9 agonist ODN2006 (InvivoGen, San Diego, CA) (34). CLL cell lines HG3, MEC1, JVM-2, and primary CLL cells were cultured in RPMI 1640, supplemented with 10% FBS, 2 mM glutamine, 100 U/ml penicillin, and 0.1 mg/ml streptomycin. For arginine starvation, cells were incubated as described (18).

### Proliferation and Cell Viability Assays

Cell proliferation was assessed by the incorporation of [^3^H]thymidine as described (18). Cell viability was determined using the FITC Annexin V Apoptosis Detection Kit I (BD Biosciences, San Diego, CA).

### Arginine Uptake

Uptake of 100 μM [^3^H]arginine (10 μCi/ml, Santa Ana, CA) was measured as described for citrulline uptake in T cells (18), except that 0.5 × 10^6^ HG3 cells or 1 × 10^6^ primary CLL cells were analyzed per well.

### Immunoblotting

Immunoblotting was performed as described (18, 35) using CAT-1 (1:5,000), y^+^LAT1 (1:3000) or y^+^LAT2 (1:10,000) antibodies produced in the laboratory of Ellen Closs, as well as ASS (Sigma Aldrich, HPA020896, 1:1,000) or GAPDH antibodies (Cell Signaling, Danvers, MA, 14C10, 1:10,000).

### Quantitative Real Time Reverse Transcription-Polymerase Chain Reaction (qRT-PCR)

Transporter mRNA expression was analyzed by two-step qRT-PCR (36). Primers and hybridization probes were previously described (35, 37) except for y^+^LAT2 (ss: 5′CACGTTCACTTACGCCAAGGT, as: 5′TCAGAGTGTCCCTGGCACAGT, probe: 5′TGCCATCATTGTCATGGGCCTTGTTA). Transporter mRNA copies were quantified using *in vitro*-synthesized RNAs, containing the complete coding region of each transporter (37).

### Lentiviral Transduction of HG3 Cells

Virus production and transduction were performed as described (38). Virus particles, carrying CAT-1 shRNA for constitutive expression were produced from HEK293T cells, cotransfected with pCMVΔR8.9, pCMV-VSV-G and pLKO.1-puro_SLC7A1-5 (shRNA: TRCN0000042967) (39) or pLKO.1-puro_SHC002 (non-target shRNA, Sigma Aldrich). 1 × 10^6^ HG3 cells (0.5 ml) were transduced with 2 ml virus supernatant. 5 h later, 1 ml of supernatant was replaced by fresh medium. One day later, transduced cells were selected by 5 μg/ml puromycin in the presence of 1 mM citrulline and 67 μM lysine tripeptide for 3 days before starting further experiments. For inducible CAT-1 knock-down, the same shRNA sequence was inserted in Tet-pLKO-puro vector (Addgene plasmid #21915) (40). Virus production was performed with HEK293T cells, cotransfected with psPAX2, pMD2.G, and Tet-pLKO-puro-sh_hCAT1 or Tet-pLKO-puro-scr as control. Transduced cells were selected by 5 μg/ml puromycin for 2 weeks before single cell clones were expanded and characterized. CAT-1 knockdown was induced by 1 μg/ml doxycycline.

### Mice

NOD.Cg-Prkdc^SCID^Il2rg^tm1Wjl^/SzJ (NSG) mice were housed under conditions according to the guidelines for animal care of the animal facility of the Johannes Gutenberg University. Experimental procedures were performed according to German regulations for the use of laboratory animals. 2.5 × 10^6^ Tet-pLKO-puro-sh_hCAT1 or Tet-pLKO-puro-scr cells were injected subcutaneously in the left flank of NSG mice. ShRNA expression was induced by 1 mg/ml doxycycline and 2% sucrose in drinking water.

### Statistical Analysis

Statistical analyses were performed with the GraphPad Prism software 6. Results are expressed as means ± SD. Statistical differences were calculated using one-way analysis of variance (ANOVA), followed by the Tukey *post-hoc* test, or using *t*-test, as appropriate. Statistical comparison of survival curves was performed with Log-rank (Mantel-Cox) test. The levels of significance were specified as ^***^p < 0.001, ^**^p < 0.01, and ^*^p < 0.05.

## Results

### Arginine Availability Is Necessary for CLL Cell Proliferation

**Figure 1:**
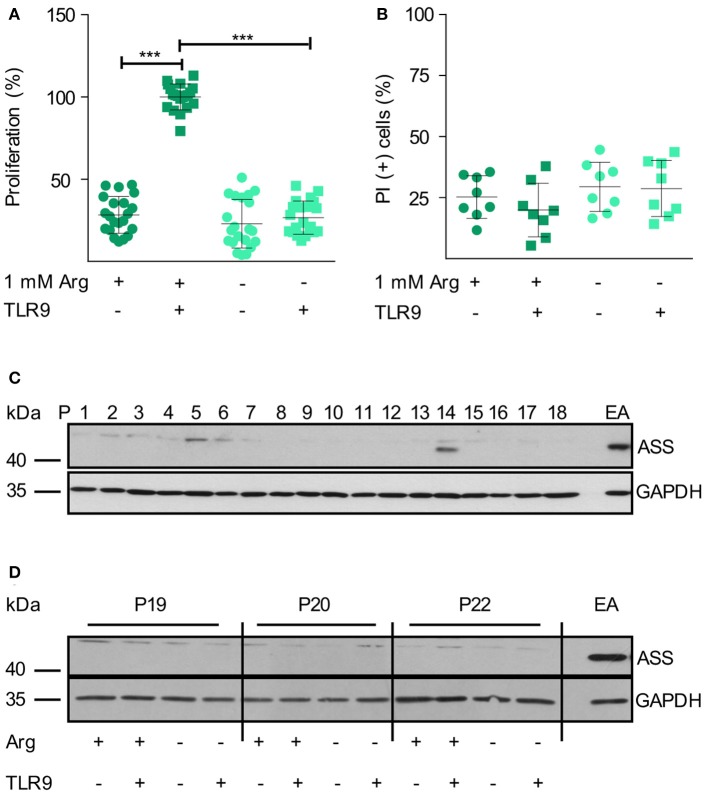
Human primary CLL cell proliferation is completely dependent on extracellular arginine. **(A,B)** Primary human CLL cells were isolated from peripheral blood of CLL patients by Ficoll density gradient centrifugation. Cells were activated with a TLR9 agonist (ODN 2006, 7.5 μg/ml) for 48 h or left unstimulated, both either in the presence (+) or absence (–) of 1 mM arginine (Arg). **(A)** Cell proliferation was determined by the incorporation of [^3^H]thymidine over 16 h. Values of stimulated cells in the presence of arginine (mean: 5,291 ± 2,668 cpm) were set as 100% (*n* = 21 from 7 independent CLL patients; P9, 14, 15, 19, 20, 24, and 25). **(B)** Cell viability: cells were stained with propidium iodide (PI) and then analyzed by flow cytometry. Values are shown as means ± SD (*n* = 8 independent donors, P9, 14, 15, 19, 20, 24, 25, and 26). Statistical calculations were performed by one way ANOVA with Tukey post-test. **(C)** ASS and Glycerinaldehyde 3-phosphate dehydrogenase (GAPDH) protein expression was analyzed by Western Blot in PB CLL cells from 18 consecutive patients (P1-18). **(D)** ASS and GAPDH protein expression were analyzed by Western Blot in PB CLL cells from 3 different patients (P19, 20, 22), cultured as described in **(A)**. EA.hy926 (EA) endothelial cells served as positive control for ASS.

We first studied the influence of arginine availability on primary human CLL cells, isolated from the peripheral blood (PB) of highly leukemic CLL patients. In CLL, the proliferating fraction is in the bone marrow and in the lymph nodes, while the cells in the blood are arrested in G_0_/G_1_ phase (34, 41), PB-derived CLL cells do therefore not proliferate *in vitro* but can be activated by surface Ig-crosslinking or by triggering TLR9 (34). Upon TLR9-mediated CLL cell activation in conventional cell culture medium containing 1 mM arginine, CLL cells entered the cell cycle and proliferation could be detected (Figure 1A). In the absence of arginine, this proliferative response was completely abolished (Figure 1A). CLL viability was not modulated by the absence of arginine within 48 h (Figure 1B).

Since ASS expression and functional arginine auxotrophy have not been studied in CLL so far, we analyzed this metabolic feature in primary PB-derived CLL cells. In CLL samples of 18 consecutive patients (Supplementary Table S1), we only saw ASS protein expression in one sample (patient 14; Figure 1C). Upon arginine depletion, tumor cells sometimes induce or upregulate ASS (20). We therefore analyzed if such a metabolic rescue strategy occurs in CLL cells. When primary CLL cells were TLR9-activated for 48 h, ASS was not induced, even under arginine depletion (Figure 1D).

**Figure 2:**
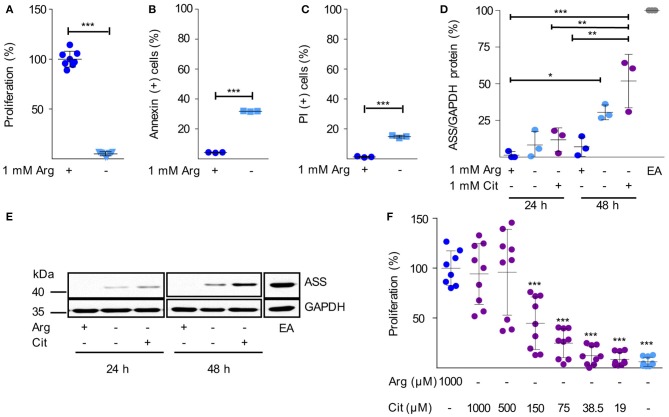
Human immortalized CLL cells do not express functionally relevant amounts of ASS and are therefore arginine auxotrophic. **(A–C)** Human HG3 CLL cells were incubated for 48 h in cell culture medium in the presence (+) or absence (–) of 1 mM arginine (Arg). **(A)** Cell proliferation was determined by the incorporation of [^3^H]thymidine over 16 h. Data are shown as means ± SD. Proliferation in the presence of arginine (mean: 65,402 ± 52,269 cpm) was set as 100% (*n* = 8–9). **(B,C)** Cells were stained with Annexin V and propidium iodide (PI) and were then analyzed by flow cytometry (*n* = 3). Values are shown as means ± SD. Statistical calculations were performed by *t*-test. **(D,E)** HG3 CLL cells were incubated for 24 and 48 h (h) in the presence (+) or absence (–) of 1 mM arginine and citrulline (Cit). ASS and GAPDH protein expression were analyzed by SDS-PAGE and Western Blot in cell lysates. **(D)** Quantitative analyses of OD values from Western Blots of 3 individual experiments depict ASS normalized to GAPDH expression and calculated as % expression of EA.hy926 cells (EA), used as positive control. Statistical calculations were performed by one way ANOVA with Tukey post-test. **(E)** A representative Western Blot of 3 independent experiments is shown. **(F)** HG3 CLL cells were incubated for 48 h in the presence or absence of 1000 μM arginine, and supplemented with different concentrations of citrulline (0–1,000 μM). Cell proliferation was determined by the incorporation of [^3^H]thymidine for further 16 h (*n* = 8–9). Data are shown as means ± SD in % of cells incubated in the presence of arginine (mean: 201,625 ± 93,045 cpm). Statistical calculations were performed by one way ANOVA with Tukey post-test.

Next, we analyzed arginine dependence and ASS expression in human HG3 CLL cells. Arginine depletion for 48 h led to a nearly complete inhibition of HG3 cell proliferation (Figure 2A) consistent with our observation in primary CLL cells (Figure 1A). In parallel, there was a significant induction of cell death as measured by Annexin V (Figure 2B) and propidium iodide (PI) staining (Figure 2C). Comparable results were seen with the CLL cell lines MEC1 and JVM-2 (Supplementary Figures S1A–F). In contrast to the primary activated CLL cells (Figure 1D), in HG3 cells a moderate, time-dependent induction of ASS was noted upon arginine depletion, both, in the presence and absence of citrulline (Figures 2D,E). These results were recapitulated in MEC1 cells (Supplementary Figures S1G,H). Corresponding to the induction of ASS, a high supraphysiological concentration of supplemented citrulline (500–1,000 μM) induced full rescue of HG3 cell proliferation in the complete absence of arginine (Figure 2F). In contrast, at lower supplemented citrulline concentrations (20–40 μM), no relevant augmentation of HG3 cell proliferation was achieved (Figure 2F). Since PB citrulline concentrations in humans are in the range of 20 μM (42), we hypothesize that even under conditions of moderate ASS expression in tumor cells, these cells can still be considered arginine-auxotrophic with full dependence on arginine import.

### CAT-1 Is the Unique Arginine Importer in CLL Cells

**Figure 3:**
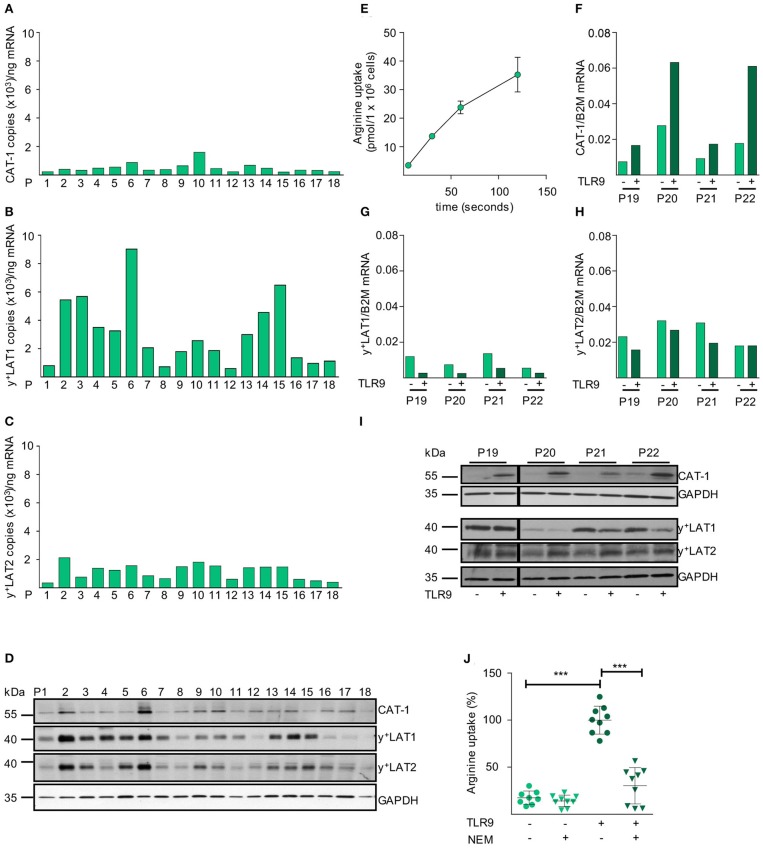
CAT-1 is the only arginine importer in primary CLL cells and its expression and arginine transport are coordinately upregulated upon activation. **(A–C)** mRNA expression of the arginine transporters **(A)** CAT-1, **(B)** y^+^LAT1, and **(C)** y^+^LAT2 was quantified by qRT-PCR in mRNA obtained from PB CLL cells of 18 individual patients (same as in Figure 1C). Depicted is the copy number of the respective mRNAs /ng total mRNA. **(D)** Protein expression of CAT-1, y^+^LAT1, y^+^LAT2, and GAPDH (as loading control) was determined by Western Blot in lysates from PB CLL cells of 18 individual patients (same as in Figure 1C). **(E)** The uptake of 100 μM [^3^H]arginine (10 μCi/ml) into PB CLL cells was assessed over 5, 30, 60, and 120 s. Arginine uptake is depicted in pmol/1 × 10^6^ cells as means ± SD of triplicates of CLL cells from P15 (other donors analyzed: P16, 18, 23). **(F–I)** PB CLL cells from 4 patients (P19-P22) were incubated for 48 h in cell culture medium in the presence (+) or absence (−) of a TLR9 agonist ODN 2006 (7.5 μg/ml). The mRNA expression of **(F)** CAT-1, **(G)** y^+^LAT1, and **(H)** y^+^LAT2 was quantified by qRT-PCR. mRNA expressions of each transporter are shown in relation to beta 2-Mikroglobulin (B2M). **(I)** Protein expression of the three transporters and GAPDH was analyzed by Western Blot. **(J)** The uptake of 100 μM [^3^H]arginine (10 μCi/ml) was determined over 30 s in the absence or presence of the CAT inhibitor NEM (200 μM) and the presence of 1 mM leucine in 48 h TLR9-stimulated or unstimulated PB CLL cells. Data are shown as means ± SD from PB CLL cells (*n* = 8) from P9, 19, and 20. The uptake in cells stimulated with TLR9 agonist in the absence of NEM (7.4 ± 1.4 pmol/10^6^ cells) was set to 100% for each patient to address intraindividual variations. Statistical calculations were performed by one way ANOVA with Tukey post-test.

After establishing the necessity of extracellular arginine availability for CLL cell proliferation, we analyzed the mRNA expression levels of all potential arginine transporters in unstimulated primary CLL cells. We saw mRNA expression of CAT-1, y^+^LAT1, and y^+^LAT2 in all 18 consecutive CLL samples from individual patients (Figures 3A–C). No mRNA expression of CAT-2A, CAT-2B, CAT-3, b^0,+^AT, or ATB^0,+^ was detectable in any patient-derived CLL cell sample (data not shown). Protein expression of CAT-1, y^+^LAT1, and y^+^LAT2 was seen in all CLL samples, though at varying amounts and with clear interindividual differences. Also, the ratio of the expression of the individual arginine transporters to each other varied between patients (Figure 3D). In these primary CLL cells we saw a time-dependent uptake of arginine (Figure 3E).

Upon TLR9-mediated activation an induction of CAT-1 mRNA was detected in all 4 tested individual CLL samples, while y^+^LAT1 mRNA was decreased and y^+^LAT2 mRNA was not affected (Figures 3F–H). Also, no mRNA expression of CAT-2A, CAT-2B, CAT-3, b^0,+^AT, or ATB^0,+^ was detectable upon stimulation (data not shown). The very prominent activation-induced expression of CAT-1 was also seen on the protein level, while y^+^LAT1 and y^+^LAT2 protein expression was only slightly decreased and increased, respectively (Figure 3I). CAT activity, identified as arginine transport insensitive to the competitive inhibition by the neutral amino acid leucine, was 6.5 ± 2.7-fold enhanced upon TLR9-mediated activation (Figure 3J). This correlated with the activation-mediated induction of CAT-1 (Figures 3F,I) and the proliferative response of the primary CLL cells (Figure 1A). The induced arginine transport was strongly inhibited by N-ethylmaleimide (NEM) that blocks CAT-, but not y^+^LAT-mediated transport (Figure 3J) (43).

**Figure 4:**
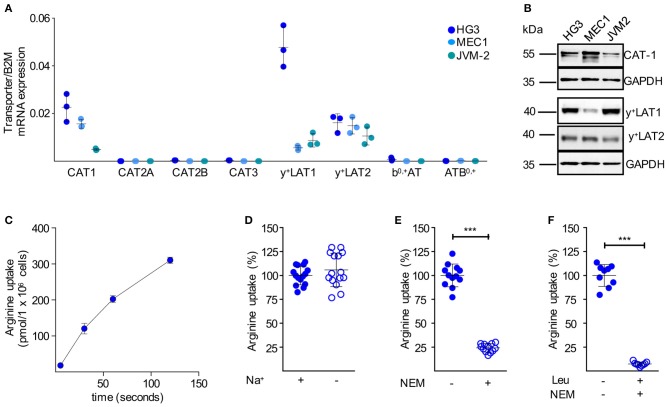
Immortalized human CLL cells take up arginine also mainly via CAT-1. **(A)** Arginine transporter mRNA expression was quantified by qRT-PCR in HG3, MEC1, and JVM-2 CLL cells in relation to beta 2-Mikroglobulin (B2M) (means ± SD, *n* = 3). **(B)** Protein expression of CAT-1, y^+^LAT1, and y^+^LAT2 was determined in HG3, MEC1, and JVM-2 cells by Western Blot. GAPDH served as loading control. One representative Western Blot of in total 3 independent experiments is shown. **(C)** Uptake of 100 μM [^3^H]arginine (10 μCi/ml) in HG3 CLL cells was assessed over 5, 30, 60, and 120 s. Arginine uptake is depicted in pmol/10^6^ cells as means ± SD (*n* = 3), shown is one representative of 3 cell batches. **(D–F)** Uptake of 100 μM [^3^H]arginine (10 μCi/ml) was determined in HG3 cells over 30 s in the absence or the presence of **(D)** sodium (*n* = 15), **(E)** 200 μM NEM (*n* = 12) or **(F)** 200 μM NEM and 1 mM leucine (*n* = 9). Arginine uptake in HG3 cells in the presence of sodium and in the absence of any inhibitor was set as 100% and means ± SD values are depicted. Statistical calculations were performed by *t*-test.

We next analyzed the mRNA expression levels of all potential arginine transporters in HG3, MEC1, and JVM-2 CLL cells and detected a comparable expression pattern to the primary cells (Figure 4A): among the CAT subfamily of arginine transporters, only CAT-1 was expressed. The arginine exporters y^+^LAT1 and y^+^LAT2 were also expressed, while no expression was detectable for b^0,+^AT and ATB^0,+^. The mRNA expression pattern was mirrored on the protein level (Figure 4B). In summary, the arginine transporter expression profile of the immortalized cell lines nicely reflected the profile in the primary human CLL cells. Arginine import into HG3 cells (Figure 4C) was about 14 times higher compared to resting human primary CLL cells (Figure 3E) (159.8 ± 86.07 vs. 11.14 ± 6.77 pmol/10^6^ cells at 30 s). Arginine uptake was independent of sodium (Figure 4D), excluding the arginine transporter system ATB^0,+^. The CAT inhibitor NEM (200 μM) reduced arginine import significantly by 75.8 ± 4.4% (Figure 4E). The addition of 1 mM leucine to NEM led to an almost complete inhibition of the arginine transport (92.7 ± 2.1%; Figure 4F). Arginine import in HG3 CLL cells was thus mainly mediated via a CAT-based transport activity, in line with our CAT1 mRNA and protein expression data (Figures 4A,B). As y^+^LATs were the only other arginine transporters expressed in HG3 cells, the minor fraction inhibited by the neutral amino acid leucine was most likely due to y^+^LAT activity. This y^+^LAT-mediated arginine uptake is only seen in the experimental setting with no competing neutral amino acids, while these transporters function mainly as arginine exporters under physiological conditions with surplus neutral amino acids in the extracellular compartment.

### Downregulation of CAT-1 in HG3 Cells Shuts Down Proliferation and Induces Cell Death

After delineating CAT-1 as the sole transmembrane arginine importer in resting and activated human CLL cells, we addressed its causative role for CLL proliferation and viability. Out of five different plasmids encoding shRNAs to downregulate CAT-1 upon lentiviral transduction, plasmid pLKO.1-puro_SLC7A1-5 maximally suppressed CAT-1 mRNA in HG3 CLL cells and was used for all further experiments. Because of the strong inhibition of HG3 cell proliferation in the absence of arginine, we anticipated an inherent inability to expand HG3 clones successfully transduced with CAT-1 shRNA, thereby lacking the potential non-redundant arginine importer. To circumvent this fundamental problem, we expanded the SLC7A1-5-transduced HG3 cells (CAT-1 k.o.) in cell culture medium supplemented with supraphysiological amounts of citrulline (1 mM), since HG3 cells upregulated ASS under intracellular arginine limitation (which should be present in the absence of CAT-1) (Figure 2E) and were able to proliferate even in the complete absence of arginine when 1 mM citrulline was substituted (Figure 2F). Since CAT-1 is also an import transporter for the amino acid lysine, we further added lysine-tripeptide to enable uptake of this amino acid via a peptide transporter (independent of CAT-1 expression).

**Figure 5:**
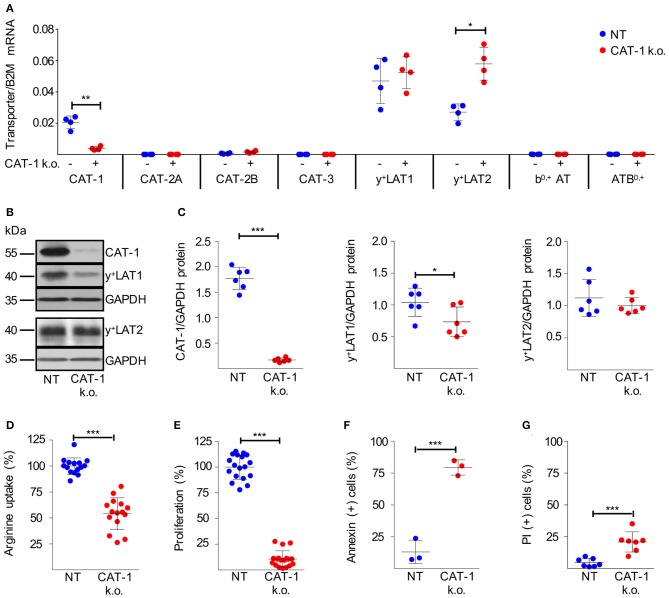
Knockdown of CAT-1 in HG3 cells inhibits proliferation and viability. **(A–G)** HG3 cells were transduced with SLC7A1-5 (+ or CAT-1 k.o.) or non-target shRNA (- or NT) lentivirus. 48 h after transduction, cells were cultured for further 48 h with puromycin (as selecting antibiotic) in the presence of 1 mM citrulline and 67 μM lysine tripeptide to allow arginine-independent expansion of CAT-1 k.o. cells. **(A)** The expression of arginine transporter mRNA was quantified in relation to beta 2-Mikroglobulin (B2M) by qRT-PCR (means ± SD, *n* = 4). **(B,C)** Protein expression of CAT-1, y^+^LAT1, y^+^LAT2, and GAPDH as loading control was analyzed in HG3 cells that were treated as described above. **(B)** One representative Western Blot is shown of 4 independent experiments. **(C)** Quantitative analysis of Western Blots. Shown are the means ± SD of transporter expression in relation to GAPDH (*n* = 6). **(D)** The uptake of 100 μM [^3^H]arginine (10 μCi/ml) was determined over 30 s in the presence of 1 mM leucine. Arginine uptake in HG3 NT cells was set as 100% (mean: 37.88 ± 11.93 pmol/10^6^ cells). Depicted are means ± SD values (*n* = 15). **(E)** Cell proliferation was determined by the incorporation of [^3^H]thymidine over 16 h (*n* = 18). Proliferation in NT HG3 cells (mean: 46,501 ± 23,722 cpm) was set as 100%. **(F,G)** Cell viability was determined by flow cytometry-based analyses of **(F)** Annexin V- (*n* = 3) and **(G)** PI- (*n* = 7) positive cells. Depicted are means ± SD values. Statistical calculations were performed by *t*-test.

The successfully expanded HG3 CAT-1 k.o. cells exhibited a suppression of CAT-1 mRNA by 80.3 ± 7.9% (Figure 5A). To study potential compensatory regulation, we analyzed mRNA expression of all arginine transporters. While the y^+^LAT1 expression level was unaffected and y^+^LAT2 mRNA was increased by 124.6 ± 76.0%, no other arginine transporter was induced as response toward CAT-1 suppression (Figure 5A). On the protein level, we realized a severe loss of the CAT-1 transporter (Figures 5B,C; 90.3 ± 3.2% decrease) upon shRNA-mediated knockdown. In contrast to the mRNA data, a slight reduction of y^+^LAT1 protein expression (28.2 ± 21.6% decrease) was detected, while y^+^LAT2 protein expression remained unaltered (Figures 5B,C). Since y^+^LAT transporters function as arginine exporters, this slight downregulation of y^+^LAT1 can be interpreted as a potential compensatory mechanism to preserve intracellular arginine levels in the context of severely reduced import, resulting from the dramatic reduction of CAT-1 transporter expression of the cells. Upon CAT-1 knockdown, HG3 cells indeed demonstrated significantly reduced system y^+^ activity, determined as leucine-insensitive arginine uptake (Figure 5D; reduction by 46.0 ± 15.4%). More importantly, the reduction of arginine import was associated with a near complete shut-down of proliferation (Figure 5E, reduction by 89.3 ± 8.4%), and an induction of cell death (Figure 5F, increase by 66.4 ± 6.5% of Annexin V-positive cells; Figure 5G, increase by 16.2 ± 7.3% of PI-stained cells) in HG3 CAT-1 k.o. cells grown in normal cell culture medium containing 1 mM arginine. These results were confirmed with HG3 cells, transduced with an alternative CAT-1 shRNA lentivirus (pLKO.1-puro_SLC7A1-1, Supplementary Figure S2). Interestingly, these results are in line with the functional impairment induced in normal, CAT-1-expressing HG3 cells cultured in medium in the complete absence of extracellular arginine (Figures 2A–C). In contrast, downregulation of y^+^LAT1 or y^+^LAT2 had no effect on cell proliferation or viability (Supplementary Figure S3). In summary, our data clearly show that CAT-1 is a non-redundant arginine importer for HG3 CLL cells: inhibition of its expression leads to a dramatic impairment of CLL proliferation and also viability.

### Downregulation of CAT-1 Inhibits CLL Tumor Growth *in vivo*

**Figure 6:**
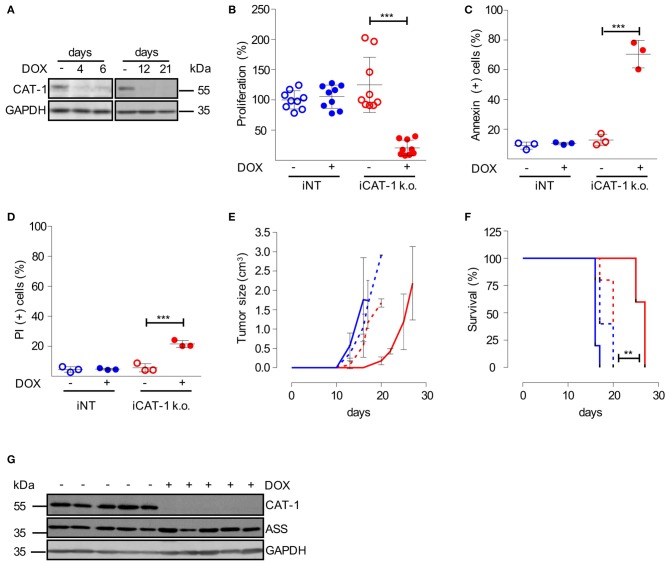
Inhibition of CAT-1 expression suppresses HG3 tumor growth *in vivo*. **(A)** HG3 cells, that had been transduced with a doxycycline-inducible shRNA CAT-1 knockdown construct (HG3_pLKO_tet_on_shCAT-1, shRNA: TRCN0000042967, termed iCAT-1 k.o.) were cultured in the absence (–) or the presence (+) of doxycycline (DOX) for 4, 6, 12 or 21 days. To allow arginine-independent expansion, cell medium was supplemented with 1 mM citrulline and 67 μM lysine tripeptide. Protein expression of CAT-1 and GAPDH was analyzed by Western Blot. **(B–D)** HG3_pLKO_tet_on_shCAT-1 (iCAT-1 k.o.) or HG3 cells transduced with an inducible non-target shRNA construct (HG3_pLKO_tet_on_non_target, iNT) were cultured for 6 days in the absence (–) or presence (+) of 1 μg/ml doxycycline and then subjected to the following analyses: **(B)** Cell proliferation was determined by the incorporation of [^3^H]thymidine for 16 h. Proliferation in HG3_pLKO_tet_on_non_target cells in the absence of doxycycline (mean: 165,043 ± 36,096 cpm) was set as 100% (*n* = 9). **(C,D)** Cells were stained with Annexin V and PI and were then analyzed by flow cytometry (*n* = 3). Values are shown as mean ± SD. **(B–D)** Statistical calculations were performed by one way ANOVA with Tukey post-test. **(E–G)** 2.5 × 10^6^ iCAT-1 k.o. (red lines) or iNT (blue lines) cells were injected s.c. in the flank of NSG mice. Mice received drinking water with (continous line) or without (dashed line) doxycycline (1 mg/ml) ad libitum (5 mice/group), depicted is one representative of two set of groups. **(E)** Tumor size was calculated based on caliper measurements (Volume = height × width^2^) and mice were sacrificed when tumor volume exceeded 3 cm^3^. **(F)** Kaplan Meier survival curves were generated. **(G)** CAT-1, ASS, and GAPDH protein expression was detected by Western Blot in explanted tumors, derived from iCAT-1 k.o. with (+) or without (−) oral administration of 1 mg/ml doxycycline via drinking water ad libitum (*n* = 5).

We finally wanted to study CAT-1 as a novel potential target for CLL therapy in an *in vivo* model. To be able to mimic a therapeutic situation, we decided to switch to an inducible CAT-1 knockdown model, in which we could start CAT-1 downregulation at a defined time point. For this model HG3 cells were transduced with a doxycycline-inducible CAT-1 lentiviral SLC7A1-5 shRNA construct and termed iCAT-1 k.o. cells. As control, HG3 cells were transduced with an inducible non-target shRNA construct and termed iNT cells. iCAT-1 k.o. cells exhibited a pronounced suppression of CAT-1 expression already 4 days after starting doxycycline treatment, and this suppression persisted to the end of the observation period of 21 days (Figure 6A). Like in the constitutive CAT-1 k.o. model (Figure 5E), we saw a strong inhibition of proliferation (Figure 6B; reduction by 82.1 ± 15.1%) and an induction of cell death (Figure 6C: increase by 57.8 ± 13.1% of Annexin V-positive cells; Figure 6D: increase by 15.9 ± 0.4% of PI-stained cells) upon doxycycline-mediated CAT-1 knockdown. We then applied these cells to a mouse CLL xenograft: after s.c. injection of HG3 CLL cells (iCAT-1 k.o. or iNT) into the left flank, mice of both tumor groups were split in a treatment group (receiving doxycycline in the drinking water), and a control group (no doxycycline), so that tumor growth was monitored in 4 different experimental groups (Figure 6E). Downregulation of CAT-1 considerably retarded tumor growth as compared to the three control groups in which tumor growth started at very similar time points (Figure 6E). The retardation of tumor growth translated into significantly (*p* = 0.0035) prolonged survival of mice (Figure 6F). After a pronounced plateau phase with completely inhibited tumor growth, there was in the end breakthrough growth with a growth kinetic that was similar to control tumors (Figure 6E). We studied potential rescue mechanisms in explanted tumors. However, CAT-1 protein expression was persistently downregulated in tumors from iCAT-1 k.o. cell-injected and doxycycline-treated mice (Figure 6G) and the expression of the other arginine transporters was at the same level over all tumor groups (Supplementary Figure S4). In contrast to HG3 cells sufficiently supplied with arginine, ASS was also expressed in tumors with normal CAT-1 expression (Figure 6G).

## Discussion

Cancer cell proliferation is coupled to the acquisition of carbon and nitrogen, that are mainly derived from non-glutamine amino acids (6), and arginine, carrying four nitrogen atoms, is a precursor for a wide range of nitrogenous compounds (3, 10). Targeting nutrient transporter proteins, as the first step fueling the rewired cancer cell metabolism, is a logical, although largely unexplored therapeutic approach (5, 44). We therefore investigated arginine transport as a potential new target to treat CLL.

We show that CLL cells fulfill the two key requirements, necessary for the successful depletion of arginine by inhibiting one specific transporter: (i) Primary CLL cells in general do not express ASS (Figures 1C,D) and are therefore arginine-auxotrophic and (ii) CLL cells non-redundantly rely on the import of arginine via one specific transporter, namely CAT-1 (Figure 5).

CLL cells share arginine auxotrophy with other lymphomas (45), acute lymphoblastic leukemia (22) and acute myeloid leukemia (21) as well as with several solid cancer entities (20). Tumor cells shut down ASS expression in order to increase their intracellular availability of the ASS substrate aspartate, which is then shuttled into nucleotide biosynthesis (46). This mostly epigenetically-driven ASS shutdown is on the one side often associated with a more aggressive clinical phenotype (45), on the other side it generates an Achilles heel of cancer cells, that could specifically be targeted by restricting arginine availability (5).

Cationic amino acid transport has been described in CLL cells already over 30 years ago (47), although the corresponding transporter was unknown at that time and had not been identified since then. In the current work we have addressed this open question in the context of a renewed interest in cancer cell metabolism and identified CAT-1 as the only arginine importer in both, CLL cell lines as well as PB CLL cells with a strong induction upon TLR9 activation in the latter. Our data are corroborated by recent findings of a coordinately induced network of genes linked to mRNA translation and amino acid metabolism, including various amino acid transporters in both, lymph node (LN) and in TLR9-stimulated PB CLL cells (34).

Only few data are published so far on the role of CAT-1 in cancer entities. Its expression has been described in blasts of patients with AML (21) and ALL (22). The siRNA-mediated knockdown of CAT-1 in both, breast cancer (48) and colorectal cancer (49) cell lines induced cell death. Upon CAT-1 knockdown, we saw a strong inhibition of CLL cell proliferation *in vitro* (Figures 5E, 6B) and of tumor growth *in vivo* (Figure 6E) without compensatory upregulation of other potential arginine import proteins or re-expression of CAT-1. The leucine-insensitive arginine uptake was less reduced than the CAT-1 mRNA and protein. This may be due to differences in the proportion of transporter localized in the plasma membrane vs. intracellular compartments. It is remarkable that an about 50% reduction in arginine uptake resulted in the almost complete inhibition of cell proliferation and pronounced cell death. This indicates a very high demand of tumor cells for arginine and suggests either transport inhibition or lowering of extracellular arginine as a promising therapeutic approach. In addition, the pronounced effect of CAT-1 down-regulation on CLL cell viability and tumor growth may partly be due to diminished uptake of the essential amino acid lysine that is also a CAT-1 substrate. However, after initial profound suppression of tumor xenograft growth, there was tumor outgrowth eventually. What might be the reason for this? Our tumor growth curves (Figure 6E) are reminiscent of the temporary arrest of tumor growth in systemically arginine-depleted animals upon application of ADI (50). In ADI-treated patients two prominent resistance mechanisms finally develop: (i) generation of neutralizing antibodies against the microbial ADI enzyme (24), and (ii) re-expression of ASS in cancer tissue with loss of arginine auxotrophy and use of ADI-generated citrulline for endogenous cancer cell arginine synthesis (20). While the former mechanism is irrelevant for our model, the ASS expression indeed detected in tumors of all experimental groups may account for the breakthrough tumor growth (Figure 6E). However, with the exception of CLL cells from one patient, PB primary human CLL cells did not express ASS even under TLR9 stimulation and arginine depletion (Figures 1C,D). We assume that the lack of ASS expression reflects the situation of proliferating CLL cells *in vivo*, because TLR9 activation induces a specific translational profile mimicking the proliferative compartment of LN CLL cells (34).

It remains to be studied how arginine restriction suppresses CLL cell proliferation and viability. In ASS-deficient prostate (51) and breast (52) cancer cells, arginine starvation induces mitochondrial dysfunction-associated autophagic cell death. In breast cancer cells, it has additionally been found that arginine is necessary for arginase II-catalyzed ornithine and subsequent polyamine synthesis to fully execute the cell cycle (53). Arginine deprivation in various ASS-negative tumor cells inhibits the Warburg effect and makes the cells more dependent on glutamine (50). In this model, combination treatment with a glutaminase inhibitor induced synthetic lethality in tumor cells (50). Another way to reduce glutamine availability is interference with its uptake (31, 32, 54–56). We therefore suggest to combine CAT-1 inhibition with strategies to reduce glutamine availability to synergistically induce tumor cell death and prevent the development of metabolic tumor escape mechanisms. Arginine deprivation through CAT-1 inhibition should render tumor cells more vulnerable also to co-applied other anti-cancer therapies. An important question is of course if the fast dividing cells of the immune system would also be affected by such a treatment. We have recently shown that primary human T cells produce endogenously sufficient amounts of arginine from citrulline for unlimited proliferation (18). This suggests that immune cells are protected from a therapeutically induced arginine deficiency. Further studies are needed to confirm a preferential damage of tumor cells by CAT-1 inhibition.

**Figure 7:**
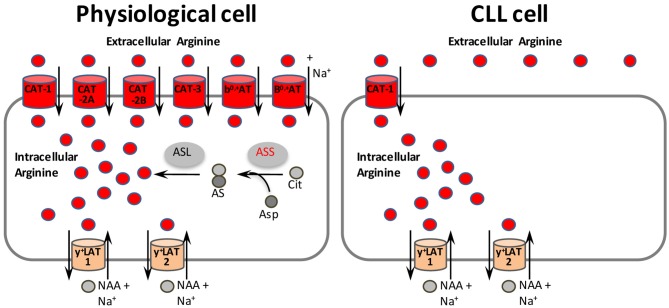
CAT-1 as novel therapeutic target structure in arginine-auxotrophic CLL cells. In physiological cells, arginine may be supplied by uptake via six different transporters (colored red, although not all the transporters listed are expressed within the same cell). The two y^+^LAT isoforms (colored orange) mediate mostly arginine export by obligatory exchange against neutral amino acids (NAA) plus Na^+^. Alternatively, arginine can be synthesized in physiological cells from citrulline and aspartate in a two step reaction with argininosuccinate synthase (ASS) as rate-limiting enzyme. In contrast, CLL cells rely solely on the uptake of arginine via CAT-1. Inhibition of CAT-1 is thus a promising new therapeutic approach for CLL and other tumor entities that share the non-redundant dependence on CAT-1 for arginine uptake. AS, argininosuccinate; ASL, argininosuccinate lyase; Asp, aspartate; Cit, citrulline.

Inhibition of amino acid transporters by small molecule compounds as a successful novel anti-cancer strategy (5) has already been reported for SLC1A5 (31), SLC7A5 (56), SLC7A11 (57), and SLC6A14 (58). An appropriate read-out assay for CAT-1 mediated arginine flux (59) could be used for compound library-based high-throughput screening to develop small molecule- or antibody-based inhibitors against CAT-1. These reagents could serve as potential novel cancer therapeutics for CLL and other arginine-auxotrophic tumor entities that share the non-redundant dependence on CAT-1 for arginine uptake (Figure 7).

## Data Availability Statement

The datasets generated for this study are available on request to the corresponding author.

## Ethics Statement

The studies involving human participants were reviewed and approved by Rhineland-Palatinate Medical Association Ethics Committee. Blood donors gave written informed consent in accordance with the Declaration of Helsinki. The patients/participants provided their written informed consent to participate in this study. The animal study was reviewed and approved by Landesuntersuchungsamt Koblenz.

## Author Contributions

AW, DP, HE, JR, KR, MT, EC, and MM contributed to the conception and/or design of the work, performed data analysis and interpretation, and revised the manuscript. AW, DP, HE, JR, EC, and MM contributed to the data acquisition. AW, EC, and MM drafted the manuscript. AW, EC, and MM finalized the article. All authors have read and approved the final manuscript and agreed to be accountable for all aspects of the work. This article contains data from the doctoral thesis of DP.

### Conflict of Interest

The authors declare that the research was conducted in the absence of any commercial or financial relationships that could be construed as a potential conflict of interest.
