# Supplementary material for: Cationic Amino Acid Transporter-1-Mediated Arginine Uptake Is Essential for Chronic Lymphocytic Leukemia Cell Proliferation and Viability
Source: Front Oncol. 2019 Nov 20;9:1268. doi: 10.3389/fonc.2019.01268 (PMC6879669; doi:10.3389/fonc.2019.01268)
Supplement: Supplementary file 1 [file Data_Sheet_1.PDF]

Pat.	Age (years)	Months since diagnosis	Lines of therapy	PB leukocytes (/ul)	LDH (U/l)[1]	Cytogenetics
1	86	52	0	62.000	226	ND
2	62	114	2	65.000	245	complex aberrant
3	71	60	1	40.000	597	del17p, trisomy12
4	69	182	8	80.000	218	ND
5	67	0	0	40.000	365	del13q, del6q, del17p
6	75	55	1	200.000	848	del13q, t(11;14)
7	75	42	0	150.000	278	ND
8	70	0	0	23.000	216	ND
9	58	0	0	9.000[2]	381	del13q, del11q, del6q, +8q
10	76	180	8	70.000	580	ND
11	70	0	0	50.000	153	normal
12	70	106	1	100.000	281	del11q, del13q
13	66	98	0	160.000	311	del13q
14	66	0	0	60.000	329	del17p
15	69	138	2	100.000	1386[3]	del13q
16	77	190	0	92.000	202	ND
17	80	54	0	74.000	419	ND
18	83	49	0	140.000	276	ND
19	59	0	0	390.000	507	del6q
20	61	0	0	200.000	477	del6q
21	44	0	0	106.000	252	del11q
22	66	67	0	105.000	717[3]	normal
23	70[4]	111	1	120.000	285	del11q, del13q
24	86	0	0	400.000	324	ND
25	56	0	0	29.000	494	trisomy 12
26	48	144	1	181.000	434	del6q, del11q, del13q

Supplementary Table S1: Key data of CLL patients.
CLL samples of 26 patients were used for this study. Key patient data at the time points of sample acquisition are listed. PB: peripheral blood. LDH: lactate dehydrogenase. ND: not done. ULN: upper limit of normal. [1]LDH ULN: 245 U/l; [2]bone marrow was used, CLL infiltration > 95%; [3]hemolytic activity was diagnosed; [4]identical to patient 12.

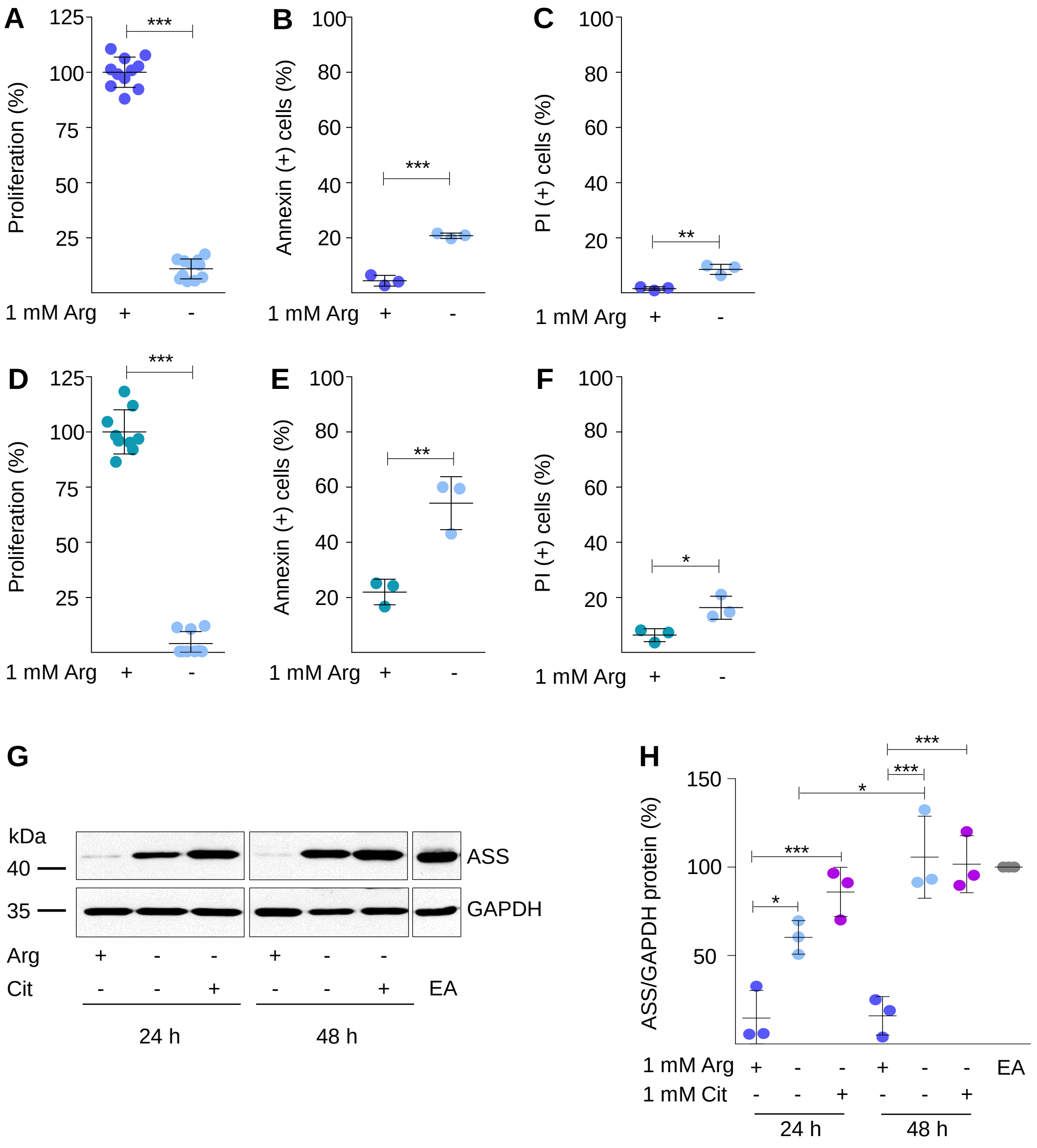

Supplementary Figure S1: Human immortalized CLL cells do not express functionally relevant amounts of ASS and are therefore arginine auxotrophic.
Human MEC1 (A-C) or JVM-2 (D-F) CLL cells were incubated for 48 hours in cell culture medium in the presence (+) or absence (-) of 1 mM arginine (Arg). (A and D) Cell proliferation was determined by the incorporation of [^{3}H]thymidine over 16 h. Data are shown as means ± SD. Proliferation in the presence of arginine (mean MEC1: 53999 ± 29148 cpm, n=11 and JVM-2: 48237 ± 36221 cpm, n=9) was set as 100%. (B, C, E, F) Cells were stained with Annexin V and propidium iodide (PI) and were then analyzed by flow cytometry (n=3). Values are shown as mean ± SD. Statistical calculations were performed by t-test. (G and H) MEC1 CLL cells were incubated for 24 and 48 hours in the presence (+) or absence (-) of 1 mM arginine and / or citrulline (Cit). ASS and GAPDH protein expression were analyzed by SDS-PAGE and Western Blot in cell lysates. (G) A representative Western Blot of in total 3 individual experiments. (H) Summarized quantitative analysis of 3 individual Western Blots: depicted are OD values of ASS normalized to GAPDH expression and calculated as % relative to the positive control EA.hy926 cells (EA). Statistical calculations were performed by one way ANOVA with Tukey post-test.

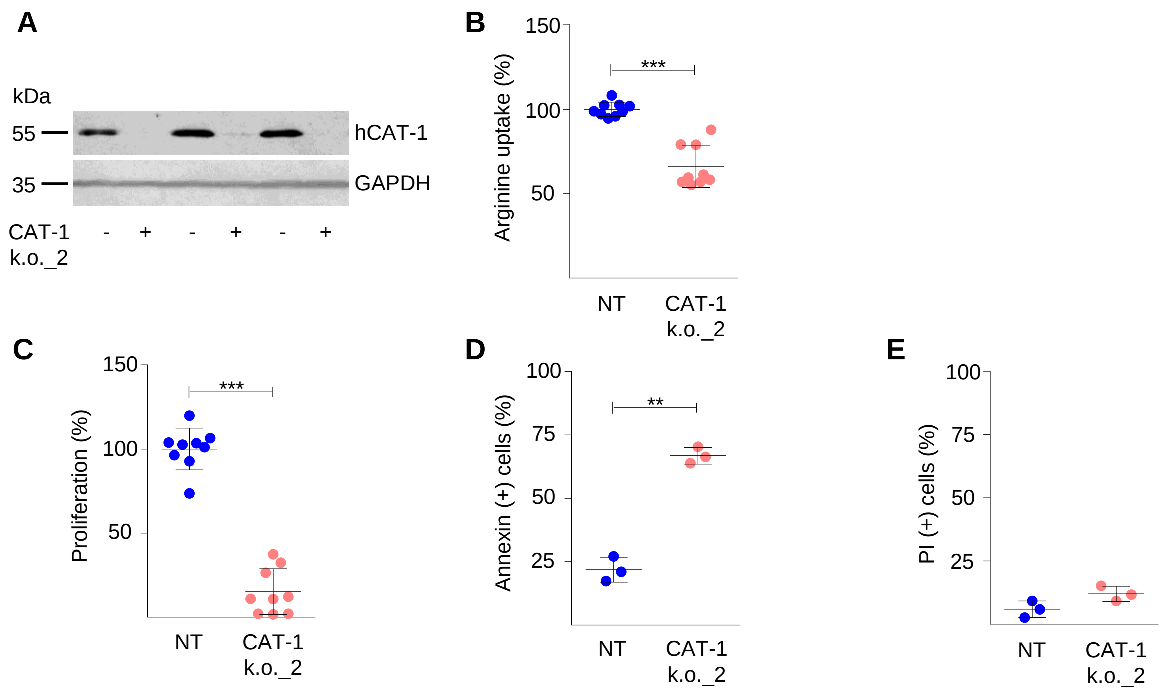

Supplementary Figure S2: Toxic effects of CAT-1 inhibition were confirmed with an alternative CAT-1 shRNA sequence.
HG3 cells were transduced with pLKO.1-puro-SLC7A1-1 (hCAT-1 k.o._2, shRNA: TRCN0000042963[1]) or pLKO.1-puro_SHC002 (NT) lentivirus. 24 hours after transduction, cells were cultured for at least further 72 hours with puromycin (as selecting antibiotic) in the presence of 1 mM citrulline and 67 µM lysine tripeptide to allow arginine-independent expansion of CAT-1 k.o. cells. (A) Protein expression of CAT-1 and GAPDH as loading control was determined by Western Blot analysis (n=3). (B) The uptake of 100 µM [^{3}H]arginine (10 µCi/ml) was determined over 30 seconds in the presence of 1 mM leucine. Arginine uptake in HG3 NT cells was set as 100% (mean: 20,95 ± 1,23 pmol/1 x 10^6 cells). (C) The cell proliferation was assessed by the incorporation of 1 µCi [^{3}H]thymidine over 16 hours (n=9). Proliferation in NT HG3 cells was set as 100% (mean: 269308 ± 39516 cpm). Cell viability was determined by flow cytometry-based analysis of (D) Annexin- or (E) PI-positive cells. Depicted are means ± SD values (n=3). Statistical calculations were performed by t-test (B-E).

References

1. Moffat J, Grueneberg DA, Yang X, et al. A lentiviral RNAi library for human and mouse genes applied to an arrayed viral high-content screen. Cell. 2006;124(6):1283-1298.

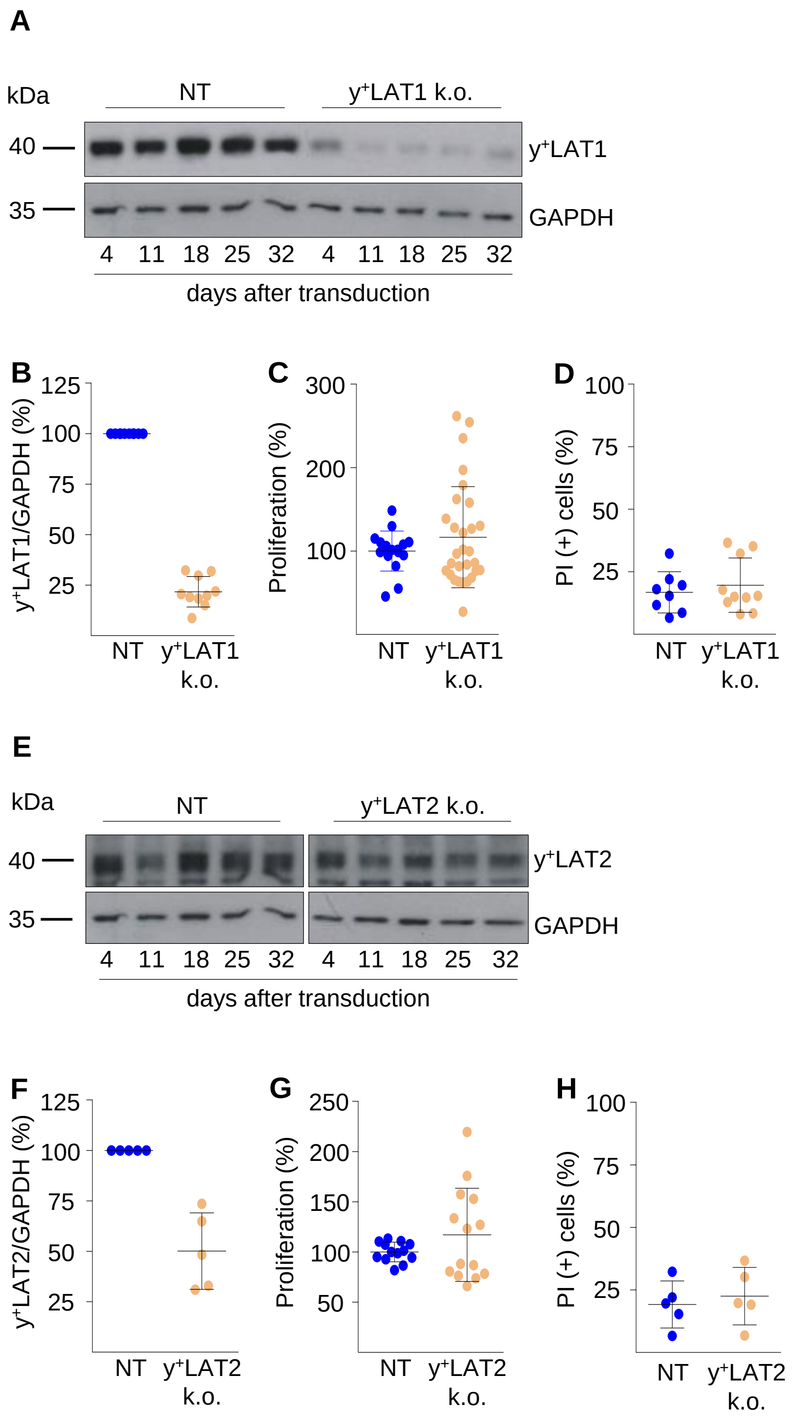

Supplementary Figure S3: Down-regulation of y+LAT1 and y+LAT2 does not affect HG3 cell proliferation and viability.
HG3 cells were transduced with pLKO.1-puro-SLC7A7-1 (y+LAT1 k.o., shRNA: TRCN0000043033, A-D)[1], pLKO.1-puro-SLC7A6-6 (y+LAT2 k.o., shRNA: TRCN0000279633, E-H)[1] or pLKO.1-puro_SHC002 (NT) lentivirus. 24 hours after transduction, cells were cultured for at least further 72 hours with puromycin (as selecting antibiotic). In contrast to CAT-1-k.o. cells, expansion of these cells was possible in normal cell culture medium. Protein expression of (A-B) y+LAT1, (E-F) y+LAT2 and GAPDH as loading control was determined by Western Blot analysis. (A,E) Shown is one representative Western Blot each. (B,F) Quantitative analysis of Western Blots. Depicted are the means ± SD of transporter expression in relation to GAPDH. Expression in NT cells was set as 100 % (y+LAT1 k.o.: n=10, y+LAT2 k.o.: n=5). (C,G) Cell proliferation was assessed by the incorporation of 1 µCi [^{3}H]thymidine over 16 hours (y+LAT1 k.o.: n=17-29, y+LAT2 k.o.: n=13-14). Proliferation in NT HG3 cells was set as 100%. (D, H) Cell viability was determined by flow cytometry-based analysis of PI-positive cells. Depicted are means ± SD values (y+LAT1 k.o.: n=8-10, y+LAT2 k.o.: n=5). Statistical calculations were performed by t-test (D, H).

References

1. Moffat J, Grueneberg DA, Yang X, et al. A lentiviral RNAi library for human and mouse genes applied to an arrayed viral high-content screen. Cell. 2006;124(6):1283-1298.

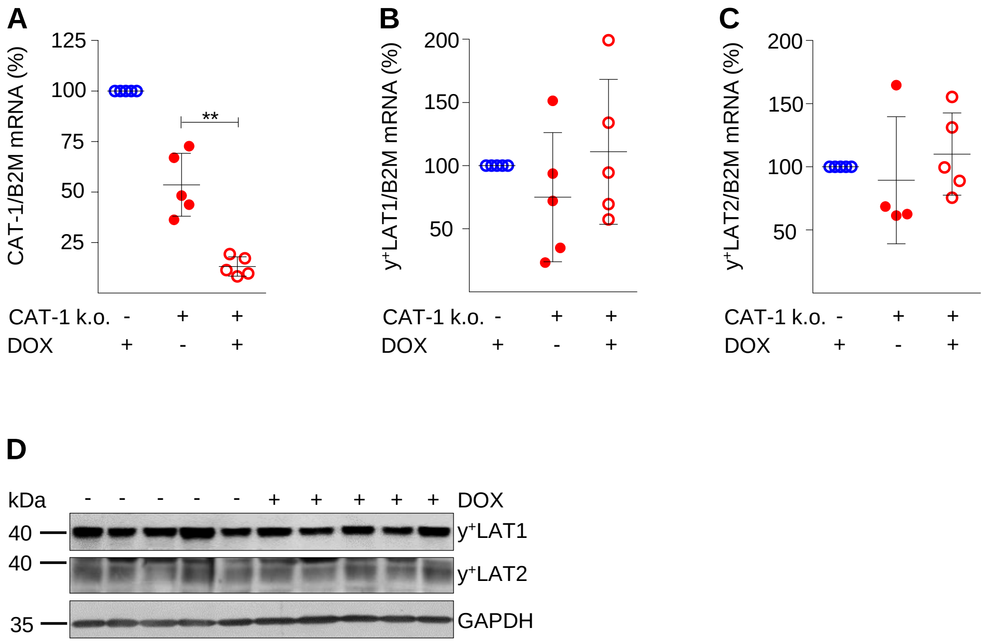

Supplementary Figure S4: Inhibition of CAT-1 expression does not change the expression of other arginine transporters.

2.5 x 10^6 HG3_pLKO_tet_on_shCAT-1 (iCAT-1 k.o.) or HG3_pLKO_tet_on_non_target cells (iNT) were injected s.c. in the flank of NSG mice. Mice received drinking water with (circles) or without (dots) DOX (1 mg/ml) ad libitum. Tumors were explanted after sacrificing and the mRNA expression of the transporters (A) CAT-1, (B) y^+LAT1 and (C) y^+LAT2 were quantified by qRT-PCR. Transporter expression is depicted as means ± SD in relation to beta 2-Mikroglobulin (B2M). The mRNA expression in HG3_pLKO_tet_on_non_target cells plus DOX was set as 100 %. The transporters CAT-2A, CAT-2B, CAT-3, $b^{0,+}$AT and $ATB^{0,+}$ were not detectable (data not shown). Statistical calculations were performed by one way ANOVA with Tukey post-test (n=4-5). (D) y^+LAT1, y^+LAT2 and GAPDH protein expression was detected in tumors derived from HG3_pLKO_tet_on_shCAT-1 cells by Western Blot (n=5).
